# GlycanGUI: Automated Glycan Annotation and Quantification Using Glucose Unit Index

**DOI:** 10.3389/fchem.2021.707382

**Published:** 2021-06-15

**Authors:** Rui Zhang, Wenjing Peng, Sakshi Gautam, Yifan Huang, Yehia Mechref, Haixu Tang

**Affiliations:** ^1^ Department of Computer Science, Luddy School of Informatics, Computing, and Engineering, Indiana University Bloomington, Bloomington, IN, United States; ^2^ Department of Chemistry and Biochemistry, Texas Tech University, Lubbock, TX, United States

**Keywords:** GUI, glycan, annotation, quantification, mass spectrometry

## Abstract

The retention time provides critical information for glycan annotation and quantification from the Liquid Chromatography Mass Spectrometry (LC-MS) data. However, the variation of the precise retention time of glycans is highly dependent on the experimental conditions such as the specific separating columns, MS instruments and/or the buffer used. This variation hampers the exploitation of retention time for the glycan annotation from LC-MS data, especially when inter-laboratory data are compared. To incorporate the retention time of glycan across experiments, Glucose Unit Index (GUI) can be computed using the dextrin ladder as internal standard. The retention time of glycans are then calibrated with respect to glucose units derived from dextrin ladders. Despite the successful application of the GUI approach, the manual calibration process is quite tedious and often error prone. In this work, we present a standalone software tool GlycanGUI, with a graphic user interface to automatically carry out the GUI-based glycan annotation/quantification and subsequent data analysis. When tested on experimental data, GlycanGUI reported accurate GUI values compared with manual calibration, and thus is ready to be used for automated glycan annotation and quantification using GUI.

## 1 Introduction

Glycosylation is a post-translational modification that plays critical roles in important biological processes such as immune response, cellular differentiation/adhesion and host-pathogen interactions (Varki et al., 2009). The aberrant alteration of glycan structure is implicit with malfunction of cells and possesses potential significance for the medical diagnosis of complex human diseases such as cancer (Ohtsubo and Marth, 2006; Pinho and Reis, 2015; Stowell et al., 2015). The Liquid Chromatography coupled Mass Spectrometry (LC-MS) is one of the most widely used techniques for glycan analysis due to its high sensitivity and throughput (Pabst and Altmann, 2011). For a chromatography, the elution order of glycans is consistent and reproducible among experiments. Hence, the retention time is often used to assess the separation of glycans and to assist glycan identification (Pabst and Altmann, 2011). However, the precise retention time of a specific glycan may vary widely depending on the experimental conditions such as the separating columns, MS instruments and/or the buffer used. This variation hampers the interpretation of LC-MS/MS data, especially when inter-laboratory data are compared without any calibrations.

To address this issue, the Glucose Unit Index (GUI) can be utilized to normalize the retention time that eliminates the variation (Mellis and Baenziger, 1981). This method employs the dextrin ladder as the internal standard and measures the retention time of glycans with respect to the glucose units derived from the dextrin ladder. The retention time is assigned to a glucose unit (GU) value, which serves as a calibration of different experiments. The GUI approach was proposed for glycomics (Campbell et al., 2008; Stockmann et al., 2013; Abrahams et al., 2018) and has been extensively used for the normalization of retention time of glycans (Ashwood et al., 2020; Gautam et al., 2020; Fabini et al., 2001; Van den Steen et al., 2006; Royle et al., 2008). In our previous study, we examined the use of permethylated dextrin for the annotation of permethylated N-glycans and their isomers derived from standard glycoproteins and human blood serum (Gautam et al., 2020). The calibrated GUI was proved to be reproducible across inter- and intra-laboratory analyses (Gautam et al., 2020).

Despite the successful application of the GUI approach, the calibration process is quite tedious, which requires manual data processing such as peak finding and curve fitting (Ashwood et al., 2019). In this paper, we present a standalone software tool GlycanGUI to automate the whole calibration process. GlycanGUI offers a graphic user interface for users to choose logarithmic or polynomial fitting that will be used in the automatic calibration of the retention time of glycan ions. Furthermore, GlycanGUI implemented the computational procedures improved from those previously developed in GlycoHybridSeq (Zhang et al., 2021) for automated glycan annotation and label-free quantification based on the total peak area of glycan ions with various charges and abducts. Therefore, GlycanGUI is ready to be used for large-scale comparative analyses of glycan abundances across many glycomic samples.

## 2 Method

### 2.1 Experimental Data

The data were obtained from the dextrin spiked human blood serum using LC-MS according to the previously published experimental protocols (Gautam et al., 2020). Briefly, 1 *μ*g of dextrin was spiked as an internal standard in initial 10 *μ*l serum before permethylation. For each injection, the released N-glycans from 1 *μ*l of the initialserum sample were resuspended in 6 *μ*l of 20% ACN and 0.1%FA. For C18 columns, solution A was 98% water, 2% ACN, and 0.1% FA and solution B was 100% ACN and 0.1% FA. The gradient started at 20% solution B and increased to 42% in 11 min. After 48 min, it reached 55% and increased to 90% in 1 min. The organic phase remained at 90% for 54 min and decreased to 20% for 6 min. The LC-MS data were acquired by using LTQ Orbitrap Velos (Thermo Scientific) instrument.

### 2.2 GlycoGUI Software

The software GlycoGUI was implemented in C# using the WPF framework for graphic user interface. After GlycoGUI reads the input LC-MS data (in thermo. raw format) using the MSFileReader library, a user can calibrate the retention time of any annotated glycans into the corresponding GUI value. The abundances (peak areas) of the glycans are computed over retention time and specifically for the detected retention range of major isomers. The source code of GlycanGUI can be found on Github at https://github.com/ruizhang84/GlycanGUIApp.

### 2.3 Glucose Units Identification

The ions corresponding to *Glucose Units* are extracted directly from the full MS spectra based on their expected mass-to-charge-ratio (m/z). Due to the instrumental noise and overlapping peaks, multiple putative glucose units (of different m/z) may be extracted at a specific retention time. To correctly label glucose units and avoid mis-identified glucose units, a dynamic programming algorithm is implemented to obtain the most likely sequence of glucose units. Briefly, a score is computed recursively for all glucose units at each retention time where a putative glucose unit (i.e., dextrin) is observed, which is based on the intensity of the peak matching the dextrin:

$$score\left[i\right]\left[u\right]=max\left(score\left[i-1\right]\left[u\right],max_{j\in \left[2,u\right]}\left(score\left[i-1\right]\left[j\right]\right)\right)$$ (1)

where *i* is the index for the observed retention time, *u* is the index of the glucose units. This equation is derived based on the fact that the higher glucose units (dextrin) always elute at a latter time than the lower units, as well as the assumption that the true peaks of the glucose units are likely more intensive than those false peaks. After identifying peaks corresponding to the glucose units in each experimental MS spectrum, the retention time with highest peak intensity for each glucose unit is used for curve fitting.

### 2.4 Curve Fitting

To calibrate the retention time of ion species into a glucose unit index (GUI), we adopt a polynomial regression,

$$y_{i}=\beta_{0}+\beta_{1}x_{i}+\beta_{2}x_{i}^{2}+\ldots +\beta_{n}x_{i}^{n}$$ (2)

where for an ion species ion *i*, $y_{i}$ is the (target) glucose unit index, $x_{i}$ is its retention time, and $\beta_{j}$ is *j*th coefficient in the polynomial regression. For a logarithmic fitting, the regression can be solved similarly by converting $x_{i}$ into $log\left(x_{i}\right)$. The linear regression can be expressed in terms of matrix multiplication for a total of *m* glucose units (i.e., dextrin ladders),

$$\left[\begin{array}{c} y_{1}\\ y_{2}\\ \ldots \\ y_{n} \end{array}\right]=\left[\begin{array}{cccccc} 1 & x_{1} & x_{1}^{2} & x_{1}^{3} & \ldots & x_{1}^{m}\\ 1 & x_{2} & x_{2}^{2} & x_{2}^{3} & \ldots & x_{2}^{m}\\ \ldots & & & & & \\ 1 & x_{n} & x_{n}^{2} & x_{n}^{3} & \ldots & x_{n}^{m} \end{array}\right]\left[\begin{array}{c} \beta_{0}\\ \beta_{1}\\ \ldots \\ \beta_{m} \end{array}\right]$$ (3)

or simply in the vector form as,

$$\vec{y}=X\vec{\beta }$$ (4)

Using the least square estimation, the coefficients are computed as,

$$\vec{\beta }={\left(X^{T}X\right)}^{-1}X\vec{y}$$ (5)

where $X^{-1}$ is the inverse of the matrix X, which can be computed by using the Gauss-Jordan method (Althoen and Mclaughlin, 1987). Once the coefficients are estimated using the elution time and the (target) GUI values of the dextrin ladders that are injected and observed in a LC-MS experiment, they can be used in the polynomial regression for calibrating the elution time of other ion species of interests (e.g., those annotated as glycans; see below), into GUI values.

### 2.5 Glycan Annotation and Label-free Quantification

We adopted the algorithm used for glycan annotation similar to one implemented in GlycoHybridSeq (Zhang et al., 2021). Briefly, the potential N-glycans are pre-computed with up to a certain maximum number of monosaccharide residues (by default, #HexNAc≤12, #Hex≤12, #Fuc≤5, and #NeuAc≤4) according to the biosynthesis rule (Zhang et al., 2021). The theoretical isotopic distribution of these N-glycans are subsequently derived using the BRAIN algorithm (Dittwald et al., 2013) based on their chemical formulas. To search for a particular N-glycan, the accurate mass value corresponding to its most abundant isotopic ion is searched against the peaks in a full MS spectrum with a given mass tolerance. The most abundant isotopic ion is expected to be most intensive and thus is most likely to be observed in an experimental spectrum if the N-glycan is indeed present in the sample. We adopted the same spectrum pre-processing procedure including charge derivation and peak picking as that used in GlycoHybridSeq. To speed up searching of peaks within a given mass tolerance, a bucket search algorithm is employed as employed in GlycoHybridSeq. After the most abundant isotopic ion is matched, we extend the peak matching of other isotopic ions to generate a matched isotopic envelope, which is then scored against the theoretical isotopic distribution as Pearson Coefficient (r)

$$score_{r}=\frac{{\sum \left(t_{i}-\bar{t}\right)}^{\text{​}}{\sum \left(o_{i}-\bar{o}\right)}^{\text{​}}}{\sqrt{{\sum {\left(t_{i}-\bar{t}\right)}^{2}{\left(o_{i}-\bar{o}\right)}^{2})}^{\text{​}}}}$$ (6)

where $t_{i}$ and $o_{i}$ are the intensities of the matched theoretical and observed isotopic ions, and $\bar{t}$ ad $\bar{o}$ are the average intensities of all peaks in the isotopic envelope. Pearson Coefficient measures the similarity of two isotopic clusters, which offer similar measures such as Cosine Similarity, but are invariant to linear transformation of data (Van Dongen and Enright, 2012). A user-defined cutoff is used (default 0.9) to reduce likely false N-glycan annotation.

To assess the abundance of a glycan, the extracted-ion chromatogram (XIC) of glycan ion is generated by GlycanGUI by searching theoretical m/z over all full-MS spectra (as described above) and measuring the corresponding peak area at each retention time. The peak area is computed from the combined intensity of top three isotope peaks, which is adopted from the concept of top three-isotopes quantification (3TIQ) algorithm (Park et al., 2016). It is reported that considering top three isotope peaks as in 3TIQ algorithm offers more sensitive results with better signal-to-noise ratio (Park et al., 2016). To detect individual XIC of glycan isomers (that have the same composition and thus theoretical m/z), the XIC peak detection algorithm reported by Aoshima et al. Aoshima et al. (2014) is adopted with customized modification to suit for glycan detection. Briefly, the point of highest intensity (i.e., apex) is first located by searching the local maximum over XIC peaks. The leftmost and rightmost neighbor of the apex are then tracked starting from the center of apex, which are defined as the peak boundary higher than (or equal to) a given cutoff (by default 50%) of apex intensity. The left and right bounds of XIC are discovered by extending the leftmost and rightmost neighbor using a local minimum algorithm. The peak area of the detected glycan isomers were summed

$$Area=\underset{left<i<right}{\sum }A_{i}$$ (7)

where $A_{i}$ is the peak area over the detected range within left and right bound in XIC.

## 3 Results and Discussion

### 3.1 GUI Calibration

GlycanGUI provides a graphic user interface that takes mass spectra data containing dextran ladder as the internal standard (as shown in Supplementary Figure S1). It determines Glucose Unit (GU) values by fitting to 3rd order polynomial (by default) or logarithmic function (Supplementary Figure S1), which allows for calibrating the retention time to the corresponding Glucose Unit Index (GUI). This calibration process involves the determination of retention time of dextrin ladders with different number of GUs (i.e., GU 2–12) followed by the polynomial (or logarithmic) curve fitting.

**FIGURE 1:**
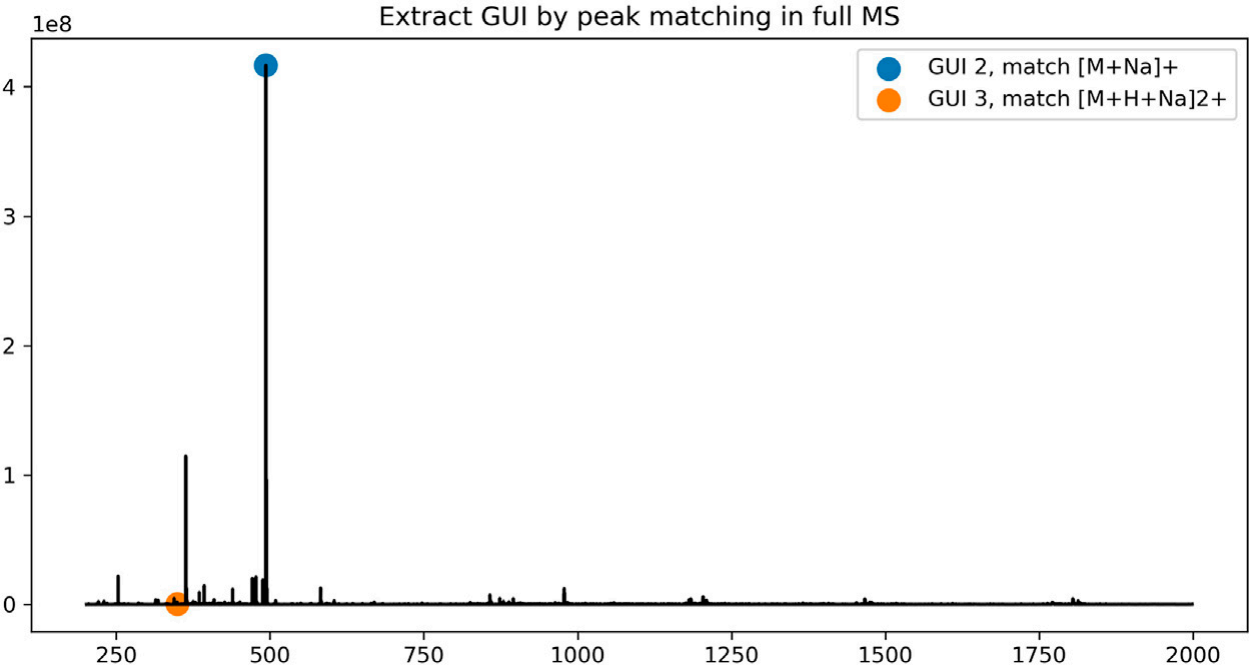
The peak assignment of dextrin ladders in a full MS spectrum.

**FIGURE 2:**
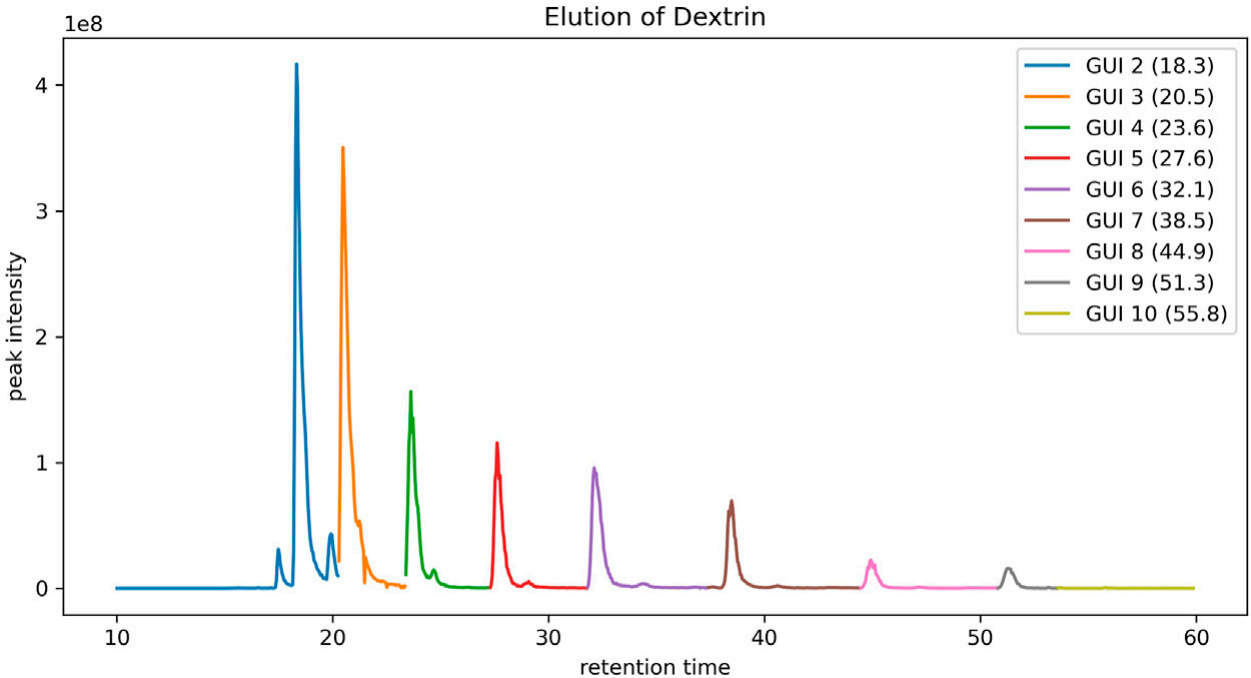
The elution profile of dextrin ladders extracted by using peak matching and the dynamic programming algorithm.

To assign the peaks of the dextrin ladders in LC-MS data, each peak in a full MS spectrum was first compared against the theoretical mass-to-charge-ratios of dextrin ladders with putative ion charges (by default up to +3) and abducts (by default the proton) using a user-defined mass tolerance. For each dextrin of a specific GU, only the most abundant isotopic ion among all matched peaks in the spectrum is recorded, along with its GU, retention time and the maximum intensity of matched peaks. However, sometimes multiple dextrin ladders with different number of GUs may be extracted from the same spectrum (Figure 1 for an example). We employed the dynamic programming algorithm as described in the Methods section to resolve the ambiguity. As shown in Figure 2, the retention time of the extracted peaks are consistent with the manual assignment in the previous study (Gautam et al., 2020) (Supplementary Figure S2), indicating GlycoGUI assigned the internal standards (dextrin ladders) accurately. Notably, for dextrins with large GUI (e.g., for GUI>10), their peak intensities become so low that are hard to be distinguished from the baseline peaks, which is difficult for manual assignment. On the other hand, the automated peak assignment by GlycoGUI can avoid such potential errors in manual assignment.

**FIGURE 3:**
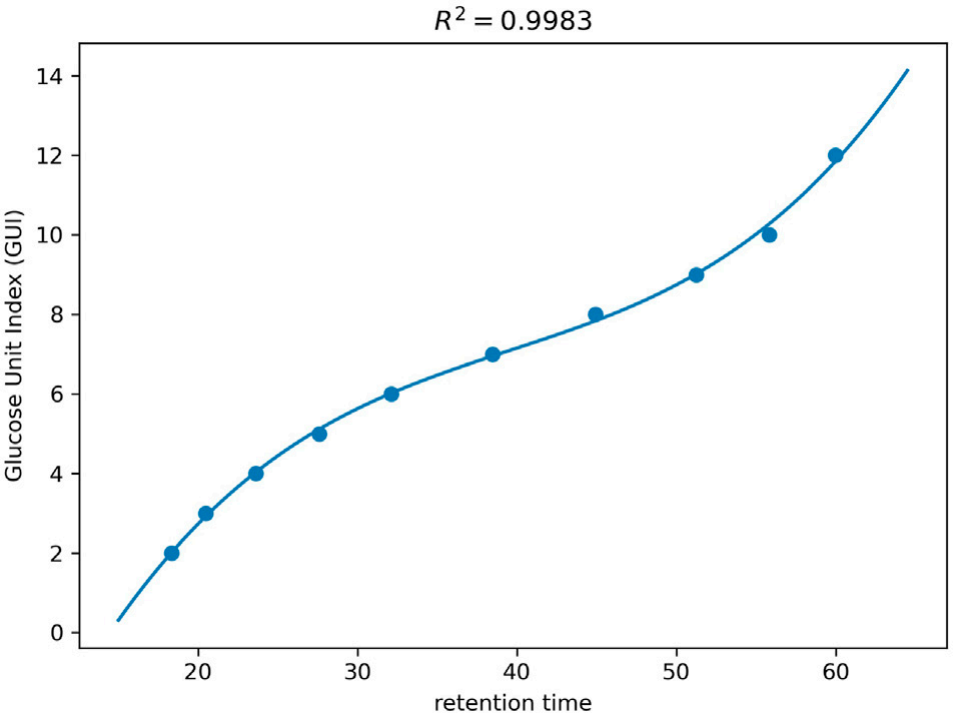
The curve fitting of GUI as a polynomial function of the retention time on dextrin ladders with different number of GUs.

To calibrate the retention time into GUI, the value of GUs (i.e., GUIs) are regressed against the retention times of the dextrin ladders (corresponding to ithe most abundant isotopic peak) using a polynomial function as described in Method. Figure 3 shows the standard plot of the GUI against the retention times for the dextrin ladders observed in a human serum sample. The coefficient of determination ($R^{2}$) is greater than 0.99 from the curve fitting, indicating the high correlation between the retention time and GUI. Alternatively, a logarithmic function can also be used for fitting the standard curve of GUIs against the retention time (as shown in Supplementary Figure S3). We implemented both curve fitting functionalities, since our previous study showed the polynomial fitting gave the best fitting results (Gautam et al., 2020), while Ashwood et al. concluded that the logarithmic fitting best described the retention profile of the dextran ladders (Ashwood et al., 2019). In our experiments, both methods received high correlation ($R^{2}>0.99$), which implies that the calibration can achieve satisfactory results by using either method, as long as the same method is consistently used for the calibrations of all samples being compared. For the results presented below, the polynomial fitting is used unless otherwise stated.

### 3.2 Glycan Annotation

To facilitate glycan identifications, glycanGUI provides a graphic user interface that allows user to annotate glycans with GUI (as shown in Supplementary Figure S4). With reported GUI of annotated glycans, the annotated glycans can be easily verified according to calibrated retention time (Ashwood et al., 2019; Gautam et al., 2020). Moreover, the validation of annotated glycans can be conducted with GUI library of known glycans (Gautam et al., 2020) to filter putative false glycan annotations.

**FIGURE 4:**
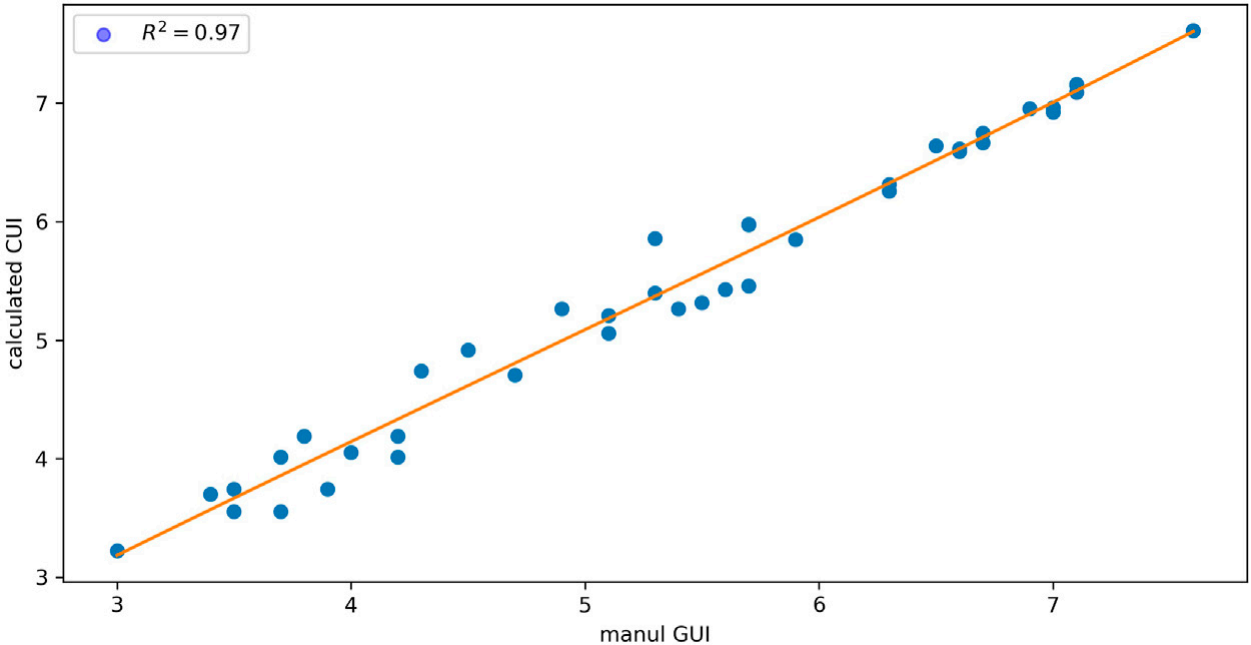
The comparison of GUI values calibrated by using GlycanGUI and by manual calibration for the N-glycans in human serum glycomic data.

To evaluate the glycan annotation and GUI calibration by GlycanGUI, the GUI values reported by GlycanGUI for the annotated N-glycans in human serum glycomic data were compared with our manual calibrations reported previously (Gautam et al., 2020). As shown in Figure 4, the calibrated GUI values by GlycanGUI is highly correlated with the manually calibrated values ($R^{2}=0.97$), indicating the automated glycan annotation and GUI calibration by GlycanGUI achieved satisfactory results. Notably, a total of 275 N-glycans were annotated by GlycanGUI, while only 51 of them were manually annotated (Gautam et al., 2020); the above comparison is performed on the N-glycans that were annotated by both methods. Given the 1,046 full MS spectra acquired in this LC-MS study, the full annotation of all N-glycans involves tremendous manual efforts. Hence, GlycanGUI serves as a reliable software tool to automate the data processing that will drastically reduces the time for data analyses.

**FIGURE 5:**
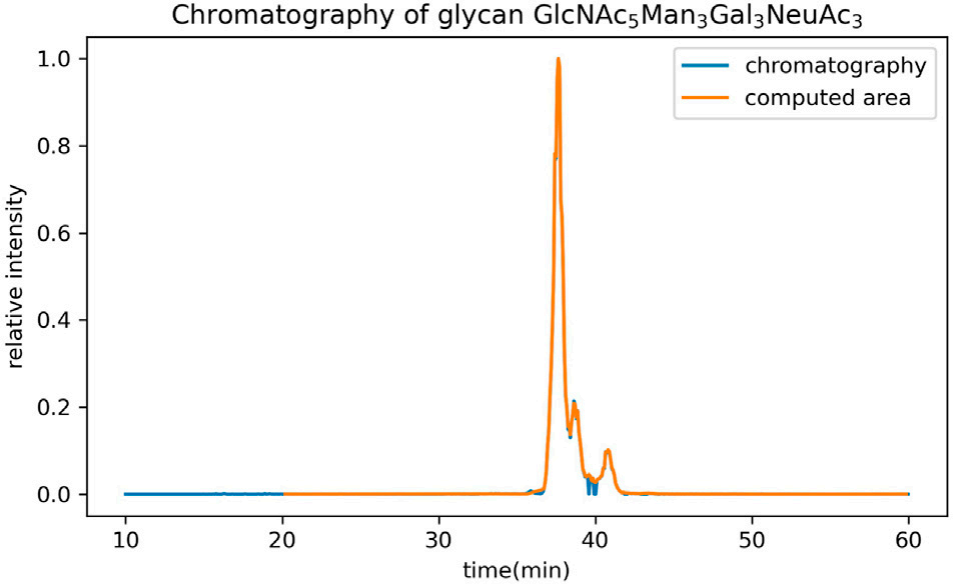
The comparison of extracted ion chromatograms reported by GlycanGUI, and by manual extraction using Xcalibur, respectively. The intensity is normalized to the highest intensity.

**FIGURE 6:**
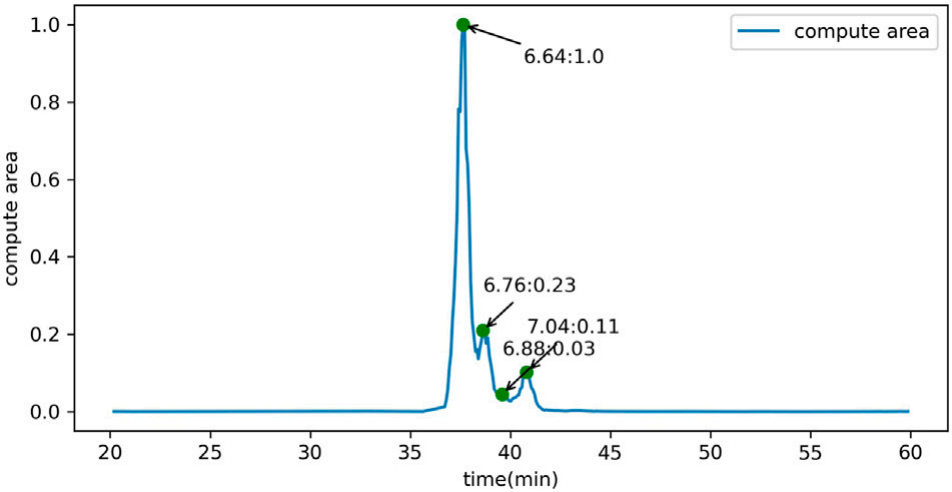
The calibration and quantification of glycan isomers distinguished by different peaks in the extracted ion chromatogram of the precursor ions with the same m/z.

**FIGURE 7:**
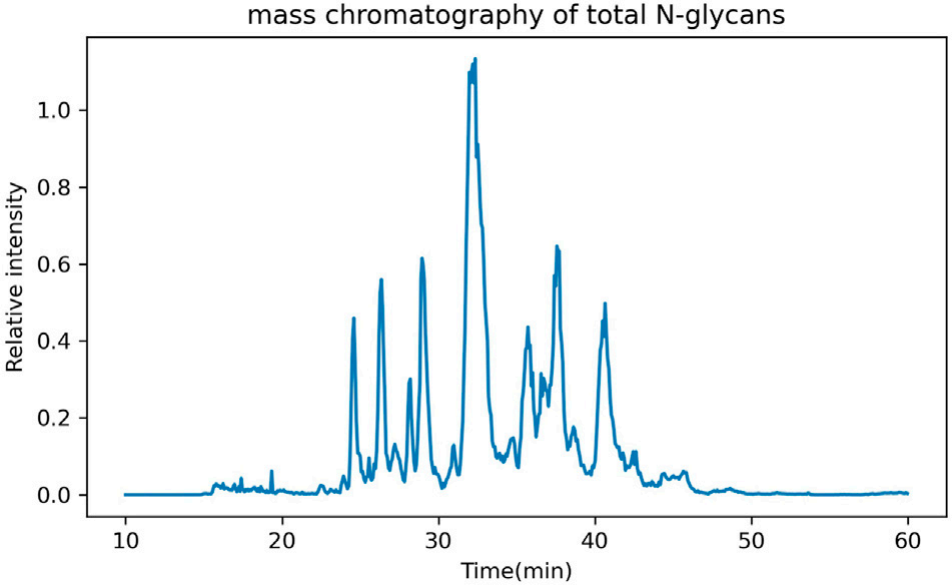
The full mass chromatography of the total N-glycans from human serum spiked dextrin.

In addition to the GUI values, GlycanGUI performs label-free quantification of the glycans with calibrated GUI values based on the peak areas in the extracted ion chromatograms (XIC). As shown in Figure 5, the XIC reported by GlycanGUI is highly correlated ($R^{2}=0.9995$) to that obtained manually using Xcalibur (see Supplementary Figure S6), indicating quantitative measurement by GlycanGUI is reliable. Moreover, GlycanGUI can automatically distinguish the glycan isomers based on the peaks in the XIC of the precursor ions with the same m/z (within the mass tolerance). The peak areas and the corresponding GUI values of the isomers are reported, respectively, as shown in Figure 6. A full mass chromatography of all the N-glycans automatically identified from human serum spiked dextrin is shown as in Figure 7. A large portion of N-glycans (60.4%) elutes during 30–40 min, while several major elution peaks are observed over 20–50 min. It is worth to mention that identified N-glycans may requires human validation, especially when interference are present. To improve glycan identification, tandem mass spectrometry (MS/MS) can be performed along with full MS so that N-glycans can be identified according to ion fragments. This improvement will be pursued in our future endeavour.

## 4 Conclusion

Glycan annotation and label-free quantification from LC-MS data often involve time consuming manual efforts. Here, we report a open-source software tool GlycanGUI for automated calibration of the elution time of glycans into GUI values and the quantification of the corresponding ions based on peak intensities. The source code of GlycanGUI is released on Github at https://github.com/ruizhang84/GlycanGUIApp. We note that although GlycanGUI has been tested extensively on glycomic datasets, the glycan annotation results should be used with caution. To produce the more reliable results for glycan annotation, GlycanGUI is preferred to be used in combination with a library of glycan GUIs (Gautam et al., 2020) or with glycan annotation software tools (Yu et al., 2013; Hu et al., 2015). In the future, we plan to implement glycan identification algorithm in GlycanGUI, for example, by exploiting the fragment ion patterns in tandem mass spectra.

## Data Availability

The original contributions presented in the study are included in the article/Supplementary Material, further inquiries can be directed to the corresponding author.
