# Supplementary material for: GlycanGUI: Automated Glycan Annotation and Quantification Using Glucose Unit Index
Source: Front Chem. 2021 Jun 15;9:707382. doi: 10.3389/fchem.2021.707382 (PMC8239159; doi:10.3389/fchem.2021.707382)
Supplement: Supplementary file 1 [file DataSheet1.PDF]

# Supplementary Material

## 1 SUPPLEMENTARY FIGURES AND TABLES

### 1.1 Figures

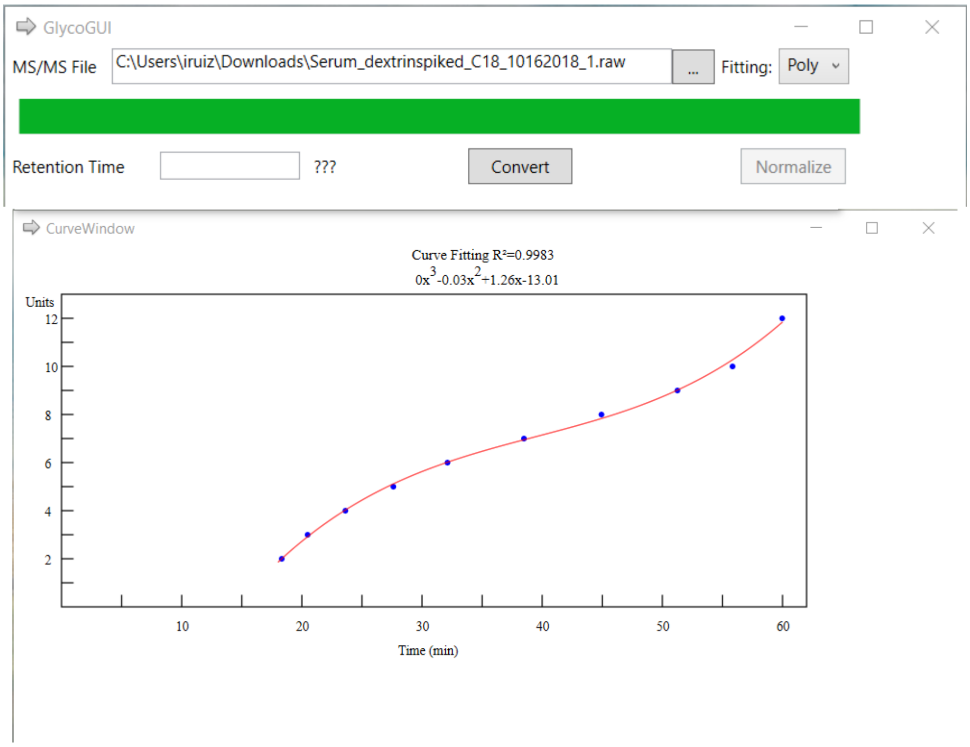


**Figure S1.** GlycanGUI interface for normalization of retention time.

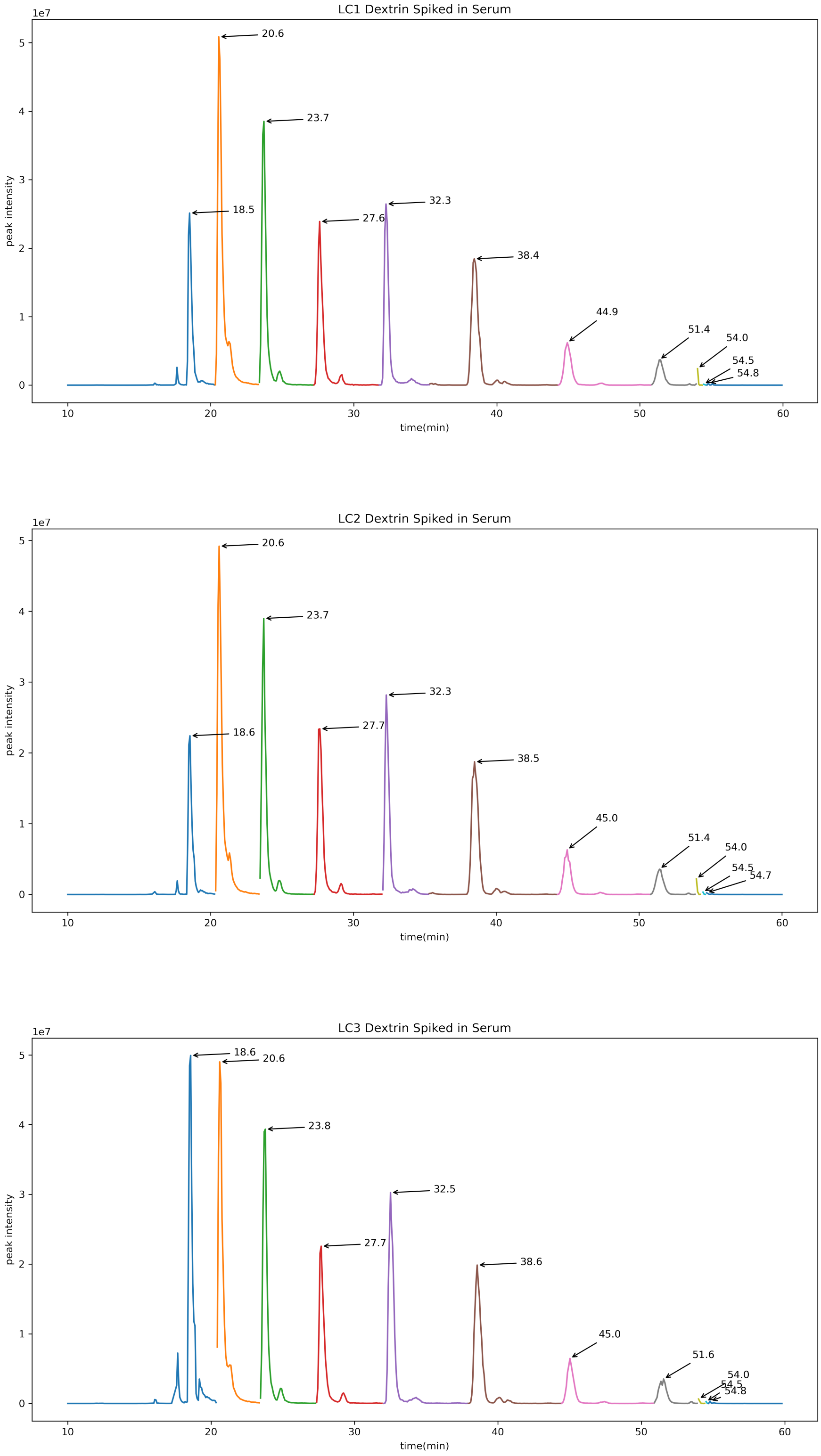


**Figure S2.** Chromatogram showing the retention time of dextrin identified by peak matching and dynamic programming.

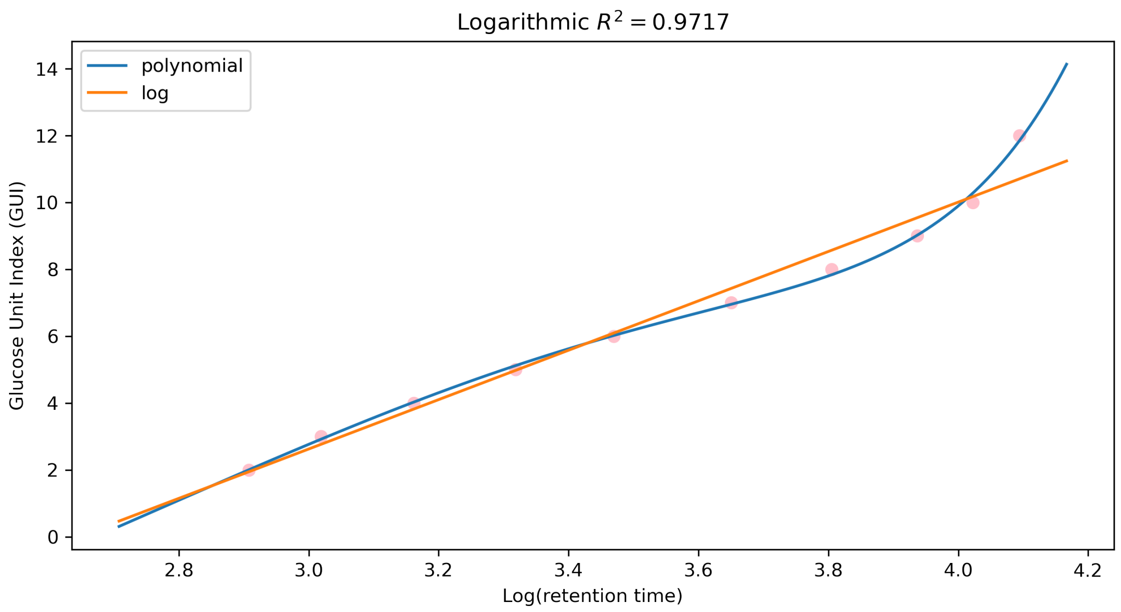


**Figure S3.** The curve fitting of GUI as a function of log retention time. The polynomial fitting is plotted in logarithmic scale to compare with that of logarithmic fitting.

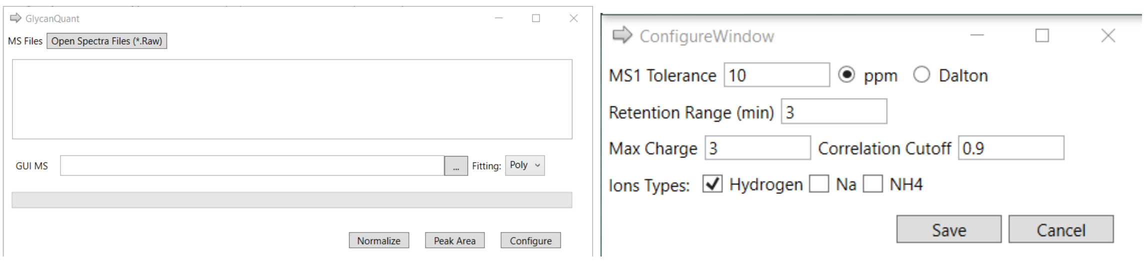


**Figure S4.** GlycanGUI interface for quantification of glycans.

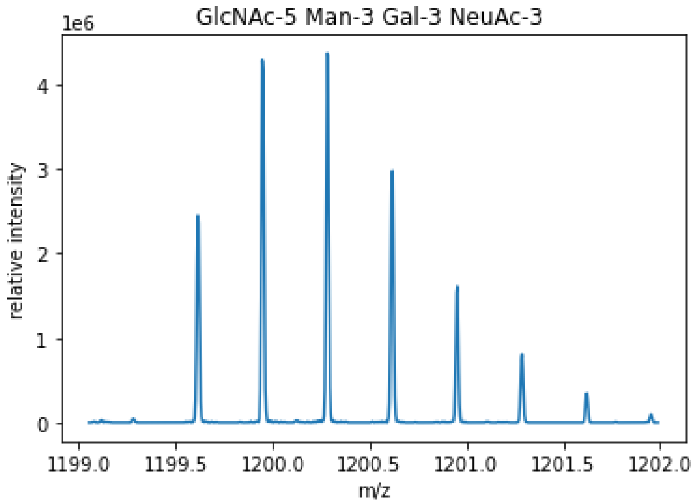


**Figure S5.** The example of an isotopic cluster of glycan $GlcNAc_5Man_3Gal_3NeuAc_3$.

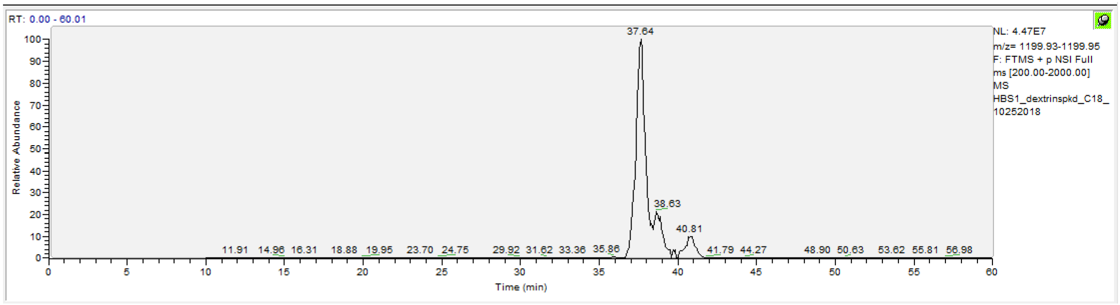


**Figure S6.** The snapshot of Xcalibur (Thermo Scientific) generated chromatograph.

## 1.2 Tables

Table S1: Glycan automatically annotated (including manual annotated were highlighted in bold.

| Glycan Annotated | GUI |
|---|---|
| $HexNAc_{10}Hex_7Fuc_2$ | 1.98,6.3,6.54,7.68 |
| $HexNAc_6Hex_4Fuc_1NeuAc_1$ | 0.59,1.37,1.67,2.84,3.33 4.16,5.78,6.31,6.63,1.23 6.56 |
| $HexNAc_6Hex_6Fuc_1$ | 0.23,1.12,2.28,2.6,3.23 3.77,4.52,5.13,5.55,6.33 6.7,7.15,5.44,6.66 |
| $HexNAc_6Hex_7Fuc_2NeuAc_2$ | 0.34,1.37,2.46,4.88,6.42 8.14,10.58 |
| $HexNAc_9Hex_9Fuc_2$ | 3.57,8.06,9.13,10.46 |
| **$HexNAc_5Hex_6NeuAc_2$** | 0.36,6.31,6.61,7.09,6.02 6.68 |
| **$HexNAc_6Hex_7Fuc_1NeuAc_3$** | 7.64,7.86,8.01 |
| $HexNAc_6Hex_7Fuc_3NeuAc_2$ | 1.36,5.24,5.98,6.74,6.99 8.01 |
| **$HexNAc_5Hex_6Fuc_1NeuAc_1$** | 0.21,1.45,6.2,6.37,6.5 6.62,7.02,1.7,6.01,6.56 |
| $HexNAc_9Hex_7Fuc_1$ | 1.29,3.67,4.06,6.74,7.67 |
| $HexNAc_6Hex_4Fuc_2$ | 0.2,0.65,1.41,1.9,2.64 3.1,3.58,4.13,4.52,4.84 5.55,6.74,14.06,1.53,5.71 6.49,10.5 |
| $HexNAc_8Hex_8Fuc_2$ | 0.72,1.74,3.83,6.42,7.97 |
| $HexNAc_6Hex_5Fuc_2$ | 0.13,0.34,0.94,1.33,3.36 3.68,4.18,4.92,5.19,5.77 6.61,1.29,6.29,6.51,7.0 |
| **$HexNAc_5Hex_4NeuAc_1$** | 0.26,1.12,1.86,4.58,4.9 5.58,6.0,0.28,1.33,4.58 5.54,5.94,6.28,9.51,10.56 |

*Continued on next page*

Table S1 – *Continued from previous page*

| **Glycan Annotated** | **GUI** |
|---|---|
| $HexNAc_{10}Hex_8Fuc_1$ | 0.2,2.2,4.37,6.27,10.54 |
| $HexNAc_{10}Hex_9Fuc_1$ | 4.08,4.87,5.29,6.65,6.98<br>8.1 |
| $HexNAc_6Hex_5Fuc_3$ | 0.22,0.76,2.13,3.38,3.57<br>3.98,4.3,4.64,5.3,5.47<br>5.83,6.24,6.81,7.68 |
| **$HexNAc_5Hex_4Fuc_1$** | 0.39,1.33,5.29,0.3,1.07<br>4.55,5.32,5.79,6.1,7.04 |
| **$HexNAc_6Hex_7NeuAc_4$** | 7.74,7.91 |
| $HexNAc_6Hex_7Fuc_5NeuAc_3$ | 8.12,9.42 |
| **$HexNAc_6Hex_7NeuAc_2$** | 0.23,2.17,2.31,5.64,6.22<br>6.8,7.18,10.49 |
| $HexNAc_5Hex_6Fuc_2NeuAc_2$ | 0.22,1.36,1.94,2.89,4.27<br>5.47,5.86,7.73,7.93,10.46<br>11.72,5.93,6.19,6.43,6.64<br>7.07,7.28 |
| $HexNAc_9Hex_9Fuc_1$ | 1.44,5.11,8.29,9.27,10.54 |
| $HexNAc_9Hex_7Fuc_3$ | 7.64,7.86,8.01 |
| $HexNAc_6Hex_6Fuc_3NeuAc_1$ | 1.24,2.35,3.02,7.63 |
| $HexNAc_4Hex_5Fuc_3NeuAc_1$ | 0.05,0.3,0.65,1.65,2.92<br>3.94,5.1,5.99,6.58,6.76<br>7.02,7.49,7.74,10.77,3.86<br>5.21,5.63,5.93,6.23,6.48<br>6.95,7.29,7.66 |
| $HexNAc_5Hex_5Fuc_2$ | 0.46,1.35,5.5,5.8,6.37<br>1.57,2.89,3.83,5.16,5.61<br>5.76,6.02,6.41,6.92,7.04 |
| $HexNAc_9Hex_9$ | 1.66,3.54,7.54,8.88 |
| $HexNAc_6Hex_5Fuc_2NeuAc_2$ | 0.76,1.96,3.47,4.28,6.08<br>6.25,10.51,2.06 |
| $HexNAc_{10}Hex_9NeuAc_1$ | 4.46,9.84 |
| $HexNAc_6Hex_5Fuc_5NeuAc_2$ | 0.24,1.16,4.01,6.11,7.86<br>8.63 |
| $HexNAc_6Hex_5Fuc_1NeuAc_1$ | 0.5,2.28,5.2,6.6,8.36<br>2.89,6.74,7.42 |
| $HexNAc_8Hex_7Fuc_3$ | 0.57,2.67,2.96,4.22,6.45<br>7.27,7.62,8.02,11.87 |
| $HexNAc_5Hex_3Fuc_4$ | 0.27,1.41,1.97,4.4,0.37<br>0.98,1.62,3.2,6.32,10.42 |
| $HexNAc_6Hex_5Fuc_3NeuAc_2$ | 0.47,1.61,2.4,4.56,6.7<br>9.93,10.58,11.42,2.67 |
| $HexNAc_6Hex_6Fuc_1NeuAc_1$ | 0.44,1.86,2.56,6.81,7.14 |

*Continued on next page*

Table S1 – *Continued from previous page*

| Glycan Annotated | GUI |
|---|---|
| | 7.1,10.51 |
| $HexNAc_8Hex_9Fuc_1$ | 0.77,1.78,6.69 |
| $HexNAc_6Hex_4Fuc_5$ | 0.0,0.2,1.16,1.57,2.28 |
| | 2.96,4.5,5.18,6.09,6.66 |
| | 7.24,7.78,6.64,7.25 |
| **$HexNAc_6Hex_7NeuAc_3$** | 7.25 |
| $HexNAc_6Hex_6Fuc_2NeuAc_2$ | 0.43,1.16,2.28,3.06,3.26 |
| | 4.15,6.2,6.48,6.72,7.07 |
| | 8.22,8.63,10.42 |
| $HexNAc_8Hex_9Fuc_1NeuAc_1$ | 1.9,4.0,4.34,4.54,5.41 |
| | 6.38,6.63,6.8,7.04 |
| $HexNAc_6Hex_6$ | 0.63,1.94,4.59,5.59,5.85 |
| | 6.15,6.48,7.04,7.25,7.77 |
| | 0.0,0.38,1.07,2.01,2.46 |
| | 3.38,4.25,4.68,5.02,5.16 |
| | 5.88,6.11,6.76,8.06 |
| **$HexNAc_3Hex_4Fuc_1$** | 0.8,1.49,2.42,2.82,4.01 |
| | 4.44,4.97,0.0,0.34 |
| $HexNAc_6Hex_5Fuc_4NeuAc_1$ | 0.28,2.92,3.98,6.27,7.32 |
| | 8.08,2.25 |
| $HexNAc_9Hex_{10}NeuAc_1$ | 9.27 |
| **$HexNAc_3Hex_3Fuc_1$** | 0.46,3.56,3.92 |
| $HexNAc_8Hex_8Fuc_1$ | 0.45,1.37,7.4,7.62 |
| **$HexNAc_5Hex_6Fuc_1NeuAc_2$** | 6.59,6.75,6.96,7.15,7.61 |
| | 6.58,7.08 |
| $HexNAc_9Hex_8Fuc_1NeuAc_1$ | 7.87,8.06,8.22 |
| $HexNAc_4Hex_4Fuc_2$ | 0.29,0.81,1.45,2.46,3.63 |
| | 4.38,5.0,5.13,5.28,5.96 |
| | 6.64,7.27,0.54,4.32 |
| $HexNAc_6Hex_3$ | 0.0,1.7,1.07,4.02,5.24 |
| | 6.09 |
| $HexNAc_{10}Hex_7Fuc_1$ | 1.23,1.41,4.66,6.08,6.74 |
| | 7.11 |
| $HexNAc_5Hex_5Fuc_2NeuAc_1$ | 0.34,1.03,1.71,5.2,5.7 |
| | 6.22,6.63,7.06,7.46,1.9 |
| | 6.32,6.62,7.04 |
| $HexNAc_6Hex_3Fuc_4$ | 0.0,0.34,1.17,2.69,3.9 |
| | 5.54,7.06,7.63,1.33,1.69 |
| $HexNAc_9Hex_8Fuc_3$ | 7.74,7.93 |
| $HexNAc_6Hex_4Fuc_4NeuAc_1$ | 0.32,1.49,2.33,2.82,4.4 |
| **$HexNAc_3Hex_3$** | 0.0,3.22,3.66 |
| $HexNAc_9Hex_8Fuc_3NeuAc_1$ | 4.89,10.05 |

*Continued on next page*

Table S1 – *Continued from previous page*

| Glycan Annotated | GUI |
|---|---|
| $HexNAc_5Hex_4Fuc_4NeuAc_1$ | 0.38,1.28,5.39,6.49,6.66 |
| | 6.38 |
| **$HexNAc_4Hex_4Fuc_1$** | 0.8,3.12,4.31,4.6,4.86 |
| | 5.38,5.78,6.68,7.18,13.52 |
| $HexNAc_4Hex_5Fuc_2$ | 0.24,0.45,1.29,4.3,4.53 |
| | 5.64,6.4,0.25,4.11,4.83 |
| | 5.02,5.66,5.98 |
| $HexNAc_9Hex_9NeuAc_1$ | 6.16,8.25,9.57,10.46 |
| $HexNAc_6Hex_3Fuc_5$ | 0.25,0.42,1.49,2.71,3.98 |
| | 5.26,5.54,6.28,6.45,6.72 |
| | 7.0,1.74,3.09,6.68 |
| **$HexNAc_5Hex_6NeuAc_3$** | 2.13,6.64,6.92,7.4,2.18 |
| $HexNAc_6Hex_7Fuc_2$ | 6.98,0.41,0.77,1.47,3.84 |
| | 4.08,4.33,4.56,4.98,5.22 |
| | 5.44,5.77,6.01,6.32,6.62 |
| | 6.85,7.16,7.71,7.9 |
| $HexNAc_6Hex_4Fuc_3$ | 0.3,0.54,1.24,2.24,2.82 |
| | 3.06,3.36,4.3,5.14,6.0 |
| | 6.96,1.57,1.9,2.89 |
| $HexNAc_8Hex_9Fuc_3$ | 1.53,4.3,7.19 |
| $HexNAc_{10}Hex_7$ | 1.21,1.74,2.77,3.29,5.94 |
| | 6.53 |
| $HexNAc_6Hex_4Fuc_5NeuAc_1$ | 0.29,2.29,3.23,5.85,6.45 |
| | 7.61,8.11,8.33,8.98,2.38 |
| | 6.51,7.02,7.91 |
| $HexNAc_7Hex_7Fuc_2$ | 0.4,1.62,3.19,4.23,5.45 |
| | 6.55,7.01,7.86,2.02,5.53 |
| **$HexNAc_4Hex_5NeuAc_2$** | 0.0,4.56,5.98,6.22,6.53 |
| | 6.81,7.7,8.48,12.94,13.3 |
| | 13.84,1.12,4.54,4.97,5.3 |
| | 6.39,6.63,7.01 |
| $HexNAc_6Hex_6Fuc_5NeuAc_1$ | 0.52,2.94,3.61,5.6 |
| $HexNAc_{10}Hex_9Fuc_4$ | 9.59,10.58 |
| $HexNAc_6Hex_7Fuc_3NeuAc_3$ | 4.52,7.44,10.38 |
| $HexNAc_6Hex_6Fuc_3NeuAc_2$ | 1.04,2.89,3.47,4.01,5.48 |
| | 5.76,6.15,6.48,6.79,6.95 |
| | 7.59,9.52,9.88 |
| $HexNAc_6Hex_5Fuc_4$ | 0.17,0.9,1.43,1.94,3.2 |
| | 3.83,4.48,4.77,5.07,5.31 |
| | 5.59,5.81,6.06,6.42,6.83 |
| | 7.16,8.12,7.03 |
| $HexNAc_9Hex_8Fuc_2$ | 2.56,7.85,8.12 |

*Continued on next page*

Table S1 – *Continued from previous page*

| Glycan Annotated | GUI |
|---|---|
| $HexNAc_{10}Hex_{11}$ | 4.9,6.06 |
| $HexNAc_5Hex_4Fuc_2NeuAc_1$ | 1.03,1.45,1.94,5.24,5.51 |
| | 6.04,0.29,1.74,2.24,2.69 |
| | 3.29,4.09,5.36,6.79 |
| $HexNAc_{10}Hex_9$ | 1.4,1.7,3.47,4.18,4.75 |
| | 6.23,7.3,7.8,8.68 |
| $HexNAc_2Hex_3$ | 0.0,2.25 |
| $HexNAc_6Hex_4NeuAc_1$ | 1.36,2.28,3.77,4.54,4.89 |
| | 5.46,5.69,6.06,6.64,7.1 |
| | 0.42,1.32,2.78,3.44,5.98 |
| | 6.43,8.63 |
| $HexNAc_{10}Hex_7Fuc_5$ | 7.02,8.7 |
| $HexNAc_6Hex_6Fuc_2NeuAc_1$ | 1.03,2.01,2.82,5.79,6.7 |
| | 6.92 |
| $HexNAc_6Hex_5Fuc_1NeuAc_2$ | 0.41,2.36,3.75,5.13,6.81 |
| | 7.58 |
| **$HexNAc_6Hex_6Fuc_1NeuAc_3$** | 0.71,2.99,6.62,7.69,7.99 |
| $HexNAc_7Hex_8Fuc_1NeuAc_1$ | 1.45,2.62,3.16,7.11,11.39 |
| $HexNAc_{10}Hex_{11}Fuc_1$ | 7.13,7.89,8.46 |
| $HexNAc_8Hex_9Fuc_3NeuAc_1$ | 8.8,10.47 |
| **$HexNAc_6Hex_7NeuAc_1$** | 0.22,1.57,2.25,2.64,6.02 |
| | 6.32,6.71,7.02,10.58,11.94 |
| $HexNAc_6Hex_6Fuc_2$ | 0.34,0.57,1.66,2.6,5.55 |
| | 6.34,6.74,7.19,2.36 |
| **$HexNAc_6Hex_7Fuc_1NeuAc_4$** | 8.39,8.63 |
| $HexNAc_9Hex_{10}Fuc_2$ | 1.97,9.08 |
| $HexNAc_6Hex_7Fuc_1$ | 0.25,1.36,2.81,3.1,3.54 |
| | 3.94,4.3,4.5,4.99,5.24 |
| | 5.51,5.83,6.18,6.68,7.03 |
| | 6.86 |
| $HexNAc_6Hex_5Fuc_1$ | 2.06,5.98,6.24,6.78,7.05 |
| | 0.28,0.67,3.47,4.0,4.36 |
| | 4.6,4.76,4.95,5.35,6.06 |
| | 6.51 |
| $HexNAc_{10}Hex_7Fuc_4$ | 1.12,6.33 |
| $HexNAc_6Hex_4Fuc_2NeuAc_1$ | 0.24,0.63,1.61,2.06,3.76 |
| | 5.74,6.98,10.48 |
| $HexNAc_5Hex_6Fuc_3NeuAc_2$ | 0.42,1.57,2.14,4.01,5.96 |
| | 6.34,6.38,6.78,12.21 |
| $HexNAc_6Hex_6Fuc_5NeuAc_2$ | 1.36,3.47,4.18,4.75,6.19 |
| | 6.6,6.86,7.4,7.85,8.71 |
| **$HexNAc_6Hex_7Fuc_1NeuAc_1$** | 1.41,2.17,2.56,5.4,5.82 |

*Continued on next page*

Table S1 – *Continued from previous page*

| Glycan Annotated | GUI |
|---|---|
| | 6.66,6.96,7.16,7.04 |
| **$HexNAc_5Hex_6Fuc_2NeuAc_3$** | 7.49,7.72,7.83,8.34,11.52 |
| $HexNAc_6Hex_6NeuAc_3$ | 1.12,2.82,7.07 |
| $HexNAc_5Hex_6Fuc_4NeuAc_3$ | 2.24,5.39,8.07,8.45,9.32 |
| $HexNAc_{10}Hex_9Fuc_3NeuAc_2$ | 12.05 |
| $HexNAc_{10}Hex_7Fuc_3$ | 1.7,2.81,6.12,6.41,7.22 |
| | 7.82,9.73 |
| $HexNAc_5Hex_5Fuc_3NeuAc_2$ | 1.05,1.49,2.31,2.96,3.21 |
| | 3.57,3.77,4.18,5.01,5.88 |
| | 6.59,7.1,9.05,9.27,2.09 |
| **$HexNAc_6Hex_4Fuc_1$** | 0.57,1.37,6.22,6.42,0.0 |
| | 0.29,1.21,1.36,2.74,4.3 |
| | 5.94 |
| $HexNAc_6Hex_6Fuc_4NeuAc_1$ | 0.29,2.28,3.54,6.59,7.36 |
| $HexNAc_6Hex_5Fuc_4NeuAc_2$ | 0.85,1.97,2.99,4.12,6.45 |
| | 7.01,7.36,8.6 |
| $HexNAc_6Hex_3Fuc_2$ | 0.38,1.41,5.07,5.92,6.53 |
| | 0.0,0.88,1.32,4.67,6.33 |
| $HexNAc_7Hex_8Fuc_2NeuAc_1$ | 0.82,7.11,7.66 |
| $HexNAc_6Hex_7Fuc_4NeuAc_2$ | 1.57,3.9,6.81,7.3,7.75 |
| | 8.94,9.96,10.57 |
| $HexNAc_6Hex_7Fuc_3$ | 0.26,0.63,1.78,2.21,3.74 |
| | 4.35,5.67,6.37,6.74,6.98 |
| | 7.32,7.59,8.04,8.92 |
| **$HexNAc_4Hex_5NeuAc_1$** | 0.26,0.98,1.49,2.05,2.43 |
| | 2.99,3.73,4.25,4.96,5.19 |
| | 5.44,6.04,6.48,7.04,8.09 |
| | 0.0,0.37,1.32,1.82,4.54 |
| | 5.06,5.5,5.65,5.8,6.11 |
| | 6.29,6.5,13.52,14.06 |
| $HexNAc_5Hex_4Fuc_3NeuAc_1$ | 0.43,1.99,2.89,5.23,5.75 |
| | 8.25,1.45,1.94 |
| $HexNAc_3Hex_3Fuc_2$ | 0.72,2.44,2.94,3.3,4.78 |
| | 5.1,5.59,6.09,0.01 |
| $HexNAc_8Hex_8$ | 0.71,2.49,3.77,5.9,6.47 |
| | 6.9,7.35 |
| $HexNAc_4Hex_5Fuc_2NeuAc_2$ | 0.67,2.51,6.86,2.78,6.96 |
| **$HexNAc_5Hex_3Fuc_1$** | 0.47,0.5,1.4,2.31,5.21 |
| | 5.48,5.74 |
| $HexNAc_5Hex_5Fuc_4NeuAc_2$ | 1.33,2.21,4.12,5.73,6.19 |
| | 6.56,6.98,7.42,7.7,11.47 |
| | 12.47 |

*Continued on next page*

Table S1 – *Continued from previous page*

| **Glycan Annotated** | **GUI** |
|---|---|
| $HexNAc_5Hex_5Fuc_2NeuAc_2$ | 0.37,1.81,2.53,2.9,4.5 |
| | 4.85,5.27,5.88,6.16,6.73 |
| | 7.08,7.57,8.92,11.43,11.62 |
| $HexNAc_6Hex_7Fuc_3NeuAc_1$ | 0.69,2.85,4.06,6.83,7.44 |
| $HexNAc_9Hex_7Fuc_2$ | 7.23 |
| $HexNAc_{10}Hex_9Fuc_3$ | 4.36,8.84 |
| $HexNAc_6Hex_7Fuc_4NeuAc_1$ | 1.35,5.91,6.54,7.28,8.01 |
| **$HexNAc_4Hex_5Fuc_1NeuAc_2$** | 0.28,1.49,6.26,6.49,6.79 |
| | 7.28,6.18,6.78,7.28 |
| $HexNAc_{10}Hex_8$ | 0.94,1.57,4.41,4.86,7.07 |
| | 7.89 |
| $HexNAc_6Hex_7Fuc_2NeuAc_3$ | 8.63,8.95 |
| $HexNAc_6Hex_5NeuAc_2$ | 0.3,1.25,1.86,4.57,5.54 |
| | 6.16,6.31,6.65,9.8 |
| $HexNAc_6Hex_6NeuAc_2$ | 0.43,2.13,2.81,5.56,6.58 |
| | 7.15 |
| $HexNAc_5Hex_6Fuc_2NeuAc_1$ | 0.3,1.61,2.05,5.17,5.46 |
| | 6.39,7.01,7.34,7.89,5.76 |
| | 5.99,6.4,6.62,7.14,7.83 |
| $HexNAc_6Hex_7Fuc_4NeuAc_3$ | 3.96,8.47,9.59 |
| $HexNAc_{10}Hex_8Fuc_3$ | 4.54,8.43,8.79,9.44 |
| **$HexNAc_6Hex_7Fuc_1NeuAc_2$** | 0.28,1.41,3.44,5.59,7.3 |
| | 7.67 |
| $HexNAc_6Hex_6Fuc_3$ | 0.0,0.34,1.04,1.77,3.74 |
| | 4.05,5.32,6.88,7.24,7.66 |
| $HexNAc_5Hex_3Fuc_3$ | 0.4,1.15,2.05,2.6,0.29 |
| | 1.2,1.94,2.28,6.59,7.42 |
| $HexNAc_6Hex_6NeuAc_1$ | 2.92,6.11,0.62,4.42,6.09 |
| | 6.4,7.48 |
| $HexNAc_8Hex_7Fuc_2$ | 7.19,7.52,7.79,7.11 |
| **$HexNAc_5Hex_5NeuAc_2$** | 0.33,1.29,2.71,2.92,3.61 |
| | 4.13,4.51,5.18,5.29,6.06 |
| | 6.62,5.98,6.43,6.75 |
| $HexNAc_6Hex_3Fuc_1$ | 0.32,1.49,5.67,0.52,2.32 |
| | 3.36,4.8,5.69,6.64 |
| $HexNAc_5Hex_6Fuc_2$ | 0.42,2.05,4.98,5.48,6.17 |
| | 6.5,6.74,1.55,5.86,6.26 |
| | 6.55,6.82 |
| $HexNAc_8Hex_8NeuAc_1$ | 0.16,2.77,3.4,4.06,4.56 |
| | 4.94,5.4,5.87,6.17,6.61 |
| | 6.97,7.41 |
| $HexNAc_{10}Hex_8Fuc_3NeuAc_1$ | 9.7 |

*Continued on next page*

Table S1 – *Continued from previous page*

| Glycan Annotated | GUI |
|---|---|
| HexNAc$_6$Hex$_3$Fuc$_3$ | 0.0,0.33,1.9,2.36,2.58<br>4.95,5.49,0.58,1.98,4.08<br>6.46 |
| HexNAc$_6$Hex$_7$ | 0.17,0.3,0.79,2.22,4.75<br>5.03,5.19,5.83,6.07,7.48<br>7.73,7.86,1.74,6.13,6.68<br>7.01 |
| HexNAc$_6$Hex$_6$Fuc$_4$NeuAc$_3$ | 3.06,7.53,9.41 |
| **HexNAc$_5$Hex$_4$Fuc$_1$NeuAc$_1$** | 0.6,5.19,5.37,6.22,6.37<br>1.11,1.86,5.26,6.28 |
| **HexNAc$_6$Hex$_7$Fuc$_2$NeuAc$_4$** | 9.27,9.7 |
| HexNAc$_9$Hex$_{10}$NeuAc$_2$ | 9.0 |
| HexNAc$_6$Hex$_5$Fuc$_5$NeuAc$_1$ | 0.76,4.65,6.41,8.8,6.98 |
| HexNAc$_6$Hex$_7$Fuc$_5$NeuAc$_1$ | 0.59,4.23,6.25 |
| HexNAc$_{10}$Hex$_8$Fuc$_2$ | 0.94,8.0 |
| HexNAc$_7$Hex$_8$Fuc$_1$ | 0.32,1.32,2.24,2.93,5.68<br>6.45,6.73,8.53,5.9,6.35<br>7.96 |
| HexNAc$_{10}$Hex$_{10}$NeuAc$_1$ | 8.12,9.53 |
| **HexNAc$_4$Hex$_5$Fuc$_1$NeuAc$_1$** | 0.0,0.24,1.1,1.92,3.06<br>3.57,4.89,5.43,5.88,6.2<br>6.52,6.74,7.34,13.99,0.26<br>1.86,5.48,5.9,6.42,6.74<br>6.93 |
| **HexNAc$_3$Hex$_4$** | 0.3,0.47,3.74,4.14,4.3<br>4.52 |
| HexNAc$_8$Hex$_8$Fuc$_2$NeuAc$_1$ | 1.61,4.83,6.28,6.92,7.27<br>7.57,8.73,9.08,9.73,10.63 |
| HexNAc$_6$Hex$_5$Fuc$_5$ | 0.71,1.16,2.35,2.96,4.4<br>5.78,6.25,7.08,7.25 |
| HexNAc$_7$Hex$_8$NeuAc$_1$ | 1.44,3.44,6.43,6.74,6.79<br>7.11 |
| HexNAc$_3$Hex$_4$Fuc$_2$NeuAc$_1$ | 0.71,1.81,6.32,0.24,0.99<br>1.36,1.82,3.06,6.08,10.95 |
| HexNAc$_4$Hex$_5$Fuc$_2$NeuAc$_1$ | 0.27,1.16,2.2,4.86,6.48<br>6.72,7.24,0.8,1.37,1.98<br>2.17,4.4,4.85,5.65,6.06<br>6.76,7.23,7.48 |
| HexNAc$_7$Hex$_8$ | 1.03,2.28,4.06,5.39,6.43<br>8.34,9.85,6.74,7.06 |
| **HexNAc$_5$Hex$_6$NeuAc$_1$** | 0.2,2.92,5.74,6.08,6.3<br>6.69,1.32,2.29,5.44,6.14 |

*Continued on next page*

Table S1 – *Continued from previous page*

| Glycan Annotated | GUI |
|---|---|
| | 6.66,6.84 |
| $HexNAc_2Hex_3Fuc_1$ | 0.0,0.34 |
| $HexNAc_6Hex_4Fuc_3NeuAc_1$ | 0.34,2.22,2.78,2.22 |
| **$HexNAc_4Hex_5$** | 0.36,4.65,5.1,5.35,5.88 |
| | 0.32,1.94 |
| $HexNAc_5Hex_6Fuc_4NeuAc_2$ | 0.24,3.06,6.56,6.95,9.22 |
| | 11.44 |
| $HexNAc_9Hex_8Fuc_2NeuAc_1$ | 8.66,8.9 |
| $HexNAc_4Hex_4Fuc_3$ | 0.22,0.58,0.9,4.85,5.22 |
| | 5.38,6.09,6.38,6.69,6.97 |
| | 7.15,0.28,0.94,4.88,5.58 |
| **$HexNAc_4Hex_3$** | 0.42,0.63,1.18,3.7,4.06 |
| | 4.25 |
| $HexNAc_8Hex_9$ | 1.45,2.21,7.0,8.7 |
| **$HexNAc_6Hex_6Fuc_1NeuAc_2$** | 0.76,2.46,6.16,7.1,7.24 |
| | 7.38,7.7 |
| **$HexNAc_5Hex_5NeuAc_1$** | 0.22,1.25,5.43,5.76,6.18 |
| | 6.47,1.82,4.42,5.77,6.08 |
| | 6.43 |
| $HexNAc_{10}Hex_8Fuc_5$ | 5.41,10.58 |
| $HexNAc_8Hex_7$ | 0.43,0.71,2.85,4.01,5.4 |
| | 6.56 |
| $HexNAc_5Hex_6Fuc_4$ | 1.92,5.03,5.32,5.54,6.33 |
| | 6.64,7.45,6.09,7.29,7.9 |
| **$HexNAc_3Hex_4Fuc_1NeuAc_1$** | 0.57,1.16,5.44,1.02,1.37 |
| | 4.3,5.36 |
| **$HexNAc_5Hex_6Fuc_1NeuAc_3$** | 6.91,7.17,7.56,6.83,11.74 |
| $HexNAc_{10}Hex_9Fuc_2$ | 4.3,4.86,6.09,6.88 |
| $HexNAc_9Hex_8Fuc_4$ | 8.4,8.63 |
| $HexNAc_6Hex_4Fuc_4$ | 0.32,0.76,0.99,1.86,2.29 |
| | 2.85,3.44,3.75,4.52,4.91 |
| | 5.22,5.86,7.2,2.33,10.58 |
| $HexNAc_5Hex_5Fuc_4NeuAc_1$ | 0.37,1.16,2.35,3.23,3.47 |
| | 5.49,6.45,6.73,7.11,7.75 |
| **$HexNAc_5Hex_5Fuc_1NeuAc_1$** | 0.57,1.51,2.92,5.94,6.45 |
| | 6.77,0.94,1.44,1.98,2.75 |
| | 5.05,5.9,6.18,6.51,6.81 |
| | 7.02,7.65 |
| $HexNAc_{10}Hex_{10}Fuc_1$ | 7.48,10.58 |
| **$HexNAc_5Hex_5$** | 0.28,0.58,5.86,0.21,3.09 |
| | 5.12,5.51,5.9 |
| $HexNAc_9Hex_9NeuAc_2$ | 1.11,7.61 |

*Continued on next page*

Table S1 – *Continued from previous page*

| Glycan Annotated | GUI |
|---|---|
| **$HexNAc_6Hex_7Fuc_3NeuAc_4$** | 9.53 |
| $HexNAc_8Hex_9Fuc_2$ | 1.98,7.58,10.37 |
| **$HexNAc_4Hex_4NeuAc_1$** | 0.88,4.48,5.08,5.57,6.02 |
| | 6.54,7.74,0.0,0.38,4.58 |
| | 4.7,5.28,5.55,6.56 |
| $HexNAc_5Hex_6$ | 0.0,0.57,1.94,5.48,6.12 |
| | 0.3,2.17,2.38,2.76,5.5 |
| | 6.04,6.75,7.08,7.47,7.61 |
| | 8.06 |
| $HexNAc_6Hex_6Fuc_2NeuAc_3$ | 0.28,3.7,4.22,6.96,7.27 |
| | 7.61,7.89,8.13,8.74 |
| $HexNAc_8Hex_9NeuAc_1$ | 1.37,2.21,6.18,6.9,7.07 |
| $HexNAc_{10}Hex_{10}$ | 6.61,7.14,8.94,9.73,10.58 |
| $HexNAc_6Hex_6Fuc_4NeuAc_2$ | 1.07,1.29,2.28,3.77,4.56 |
| | 5.18,5.91,6.1,6.4,6.66 |
| | 6.91,7.19,7.5,7.83,8.3 |
| | 9.22,10.54 |
| $HexNAc_4Hex_3Fuc_2$ | 0.3,0.72,1.21,1.59,2.19 |
| | 4.59,0.77 |
| $HexNAc_9Hex_8$ | 1.16,3.21,3.67,4.4,7.99 |
| $HexNAc_7Hex_8Fuc_2$ | 0.22,2.01,2.43,3.92,6.32 |
| | 6.66,7.09,7.36,9.84 |
| $HexNAc_5Hex_6Fuc_3$ | 0.77,1.15,6.02,6.37,6.78 |
| | 5.08,6.6,7.13,8.27 |
| $HexNAc_{10}Hex_8Fuc_2NeuAc_1$ | 8.31,8.79 |
| $HexNAc_9Hex_{10}$ | 2.92,7.89 |
| **$HexNAc_4Hex_5Fuc_1$** | 0.42,1.61,4.8,5.1,5.29 |
| | 5.5,5.75,6.09,6.31,0.54 |
| | 4.65,5.17 |
| $HexNAc_4Hex_5Fuc_3$ | 0.3,1.07,2.06,4.59,4.85 |
| | 5.18,5.4,5.9,6.66,6.92 |
| | 7.66,0.21,1.29,1.78,2.96 |
| | 4.52,5.05,5.49,6.28,6.51 |
| $HexNAc_9Hex_{10}Fuc_1$ | 2.1,8.35 |
| $HexNAc_5Hex_4Fuc_2$ | 0.38,0.81,1.94,4.83,5.38 |
| | 0.16,1.25,3.54,5.13,6.38 |
| $HexNAc_4Hex_4Fuc_3NeuAc_1$ | 0.38,1.41,2.1,2.67,2.85 |
| | 3.09,3.3,4.3,4.5,5.27 |
| | 5.51,5.79,6.19,6.42,6.97 |
| | 0.36,1.2,2.01,2.24,5.55 |
| | 6.29,6.76,7.04,7.52,7.79 |
| **$HexNAc_5Hex_4$** | 0.34,0.49,1.65,5.26,5.62 |

*Continued on next page*

Table S1 – *Continued from previous page*

| Glycan Annotated | GUI |
|---|---|
| | 5.87,6.77,0.28,0.72,5.38 |
| $HexNAc_4Hex_3Fuc_3$ | 0.81,1.11,4.01,4.9,5.95 |
| | 7.01,0.17 |
| $HexNAc_{11}Hex_{11}Fuc_2$ | 11.83 |
| **$HexNAc_5Hex_5Fuc_1NeuAc_2$** | 5.46,6.41,6.81,0.2,0.68 |
| | 1.78,2.24,5.58,5.95,6.5 |
| | 6.84,7.0,7.27,7.59 |
| $HexNAc_9Hex_9Fuc_1NeuAc_2$ | 6.4,8.8,11.59 |
| $HexNAc_9Hex_8Fuc_1$ | 7.44 |
| **$HexNAc_5Hex_6Fuc_1$** | 0.76,1.73,5.85,6.17,6.56 |
| | 8.87,0.53,2.6,4.67,6.13 |
| $HexNAc_{10}Hex_8Fuc_4$ | 6.71,8.31 |
| $HexNAc_6Hex_7Fuc_2NeuAc_1$ | 0.59,1.16,6.11,6.58,7.04 |
| $HexNAc_6Hex_7Fuc_5NeuAc_2$ | 4.58,7.0,7.27,9.73 |
| $HexNAc_9Hex_9Fuc_1NeuAc_1$ | 3.67,8.93,10.5 |
| $HexNAc_6Hex_6Fuc_4$ | 0.52,2.88,4.18,5.8,6.58 |
| | 6.88,7.11,10.42 |
| $HexNAc_8Hex_7Fuc_1$ | 0.71,1.53,1.82,4.8,6.93 |
| | 7.18,7.54,6.74 |
| **$HexNAc_4Hex_4$** | 0.28,0.63,1.12,1.91,2.35 |
| | 4.19,4.58,4.91,5.04,5.34 |
| | 5.98,0.0 |
| $HexNAc_{10}Hex_{11}Fuc_1NeuAc_2$ | 10.54 |
| $HexNAc_8Hex_9NeuAc_2$ | 6.51,7.59,9.54 |
| $HexNAc_{10}Hex_{10}Fuc_5NeuAc_1$ | 12.16 |
| $HexNAc_6Hex_5$ | 0.0,0.35,1.08,2.16,2.51 |
| | 3.08,5.9,6.19,0.54,1.4 |
| | 2.49,5.68,6.28,6.64,10.44 |
| $HexNAc_5Hex_4Fuc_3$ | 0.5,2.77,3.7,5.27,5.76 |
| | 6.98,1.33,4.01,5.79,6.97 |
| $HexNAc_8Hex_9Fuc_1NeuAc_2$ | 4.76,8.92 |
| $HexNAc_5Hex_4Fuc_4$ | 0.99,2.21,6.4,6.67,6.88 |
| | 0.59,1.69,2.77,3.8,6.6 |
| $HexNAc_5Hex_6Fuc_4NeuAc_1$ | 0.68,2.54,5.23,5.65,6.2 |
| | 6.72,7.09,7.73,2.1,6.03 |
| **$HexNAc_4Hex_3Fuc_1$** | 0.0,0.67,1.06,1.82,2.57 |
| | 3.36,4.06,4.38,4.78,5.08 |
| | 5.41,7.71,13.5,14.17 |
| **$HexNAc_3Hex_4NeuAc_1$** | 0.0,0.9,4.72,0.32,1.02 |
| | 1.33,1.62,2.15,4.74,5.04 |
| | 5.21,6.21,6.5,6.89,7.48 |
| | 7.76 |

*Continued on next page*

Table S1 – *Continued from previous page*

| Glycan Annotated | GUI |
|---|---|
| **$HexNAc_5Hex_3$** | 0.0,0.18,1.16,4.9,0.18<br>1.61,2.66,3.04,4.92,5.14 |
| $HexNAc_5Hex_6Fuc_3NeuAc_3$ | 0.26,2.2,5.99,7.49,8.36<br>9.27 |
| $HexNAc_6Hex_5Fuc_2NeuAc_1$ | 1.79,6.99,1.65 |
| $HexNAc_{10}Hex_8NeuAc_1$ | 5.75,8.95 |
| $HexNAc_7Hex_7$ | 0.38,0.8,1.61,2.31,5.1<br>5.36,5.58,5.97,6.35,6.81<br>1.78,6.43,6.86,7.9 |
| $HexNAc_5Hex_5Fuc_4$ | 0.29,1.57,2.28,2.58,5.76<br>6.32,7.11,3.47,6.81,8.66 |
| $HexNAc_6Hex_6Fuc_5$ | 0.17,0.37,1.44,2.53,3.47<br>4.91,6.11,6.45,6.98,7.55<br>7.82,6.78 |
| $HexNAc_9Hex_9Fuc_3$ | 2.77,8.82 |
| $HexNAc_6Hex_6Fuc_5NeuAc_3$ | 6.65,10.42 |
| $HexNAc_{10}Hex_9Fuc_1NeuAc_1$ | 9.01 |
| $HexNAc_8Hex_8Fuc_3NeuAc_1$ | 4.52,7.05,7.48,9.8,10.58 |
| $HexNAc_4Hex_4Fuc_2NeuAc_1$ | 0.22,0.67,1.96,2.34,3.36<br>4.61,5.75,7.21,7.88,1.12<br>4.56,6.26,6.68,9.81 |
| $HexNAc_9Hex_7$ | 0.32,2.24,4.85,7.04,7.27 |
| $HexNAc_5Hex_3Fuc_2$ | 0.39,1.41,2.71,0.5,1.03<br>2.42,6.67 |
| $HexNAc_9Hex_7Fuc_4$ | 7.18,7.52,8.63,8.95 |
| $HexNAc_6Hex_7Fuc_5$ | 0.57,1.07,6.54,7.06,7.28<br>8.68 |
| $HexNAc_5Hex_6Fuc_3NeuAc_1$ | 0.32,1.29,3.16,4.42,5.11<br>6.06,7.77,8.49,6.5,7.18<br>8.65 |
| $HexNAc_{10}Hex_8Fuc_1NeuAc_1$ | 5.05,6.74 |
| $HexNAc_4Hex_5Fuc_3NeuAc_2$ | 0.27,6.63,6.8 |
| $HexNAc_6Hex_7Fuc_4$ | 0.3,1.12,1.45,2.53,3.0<br>5.14,5.75,6.18,6.37,6.62<br>6.97,7.4,7.71 |
| $HexNAc_6Hex_5NeuAc_1$ | 0.4,1.66,2.13,4.04,4.73<br>5.52,6.7,1.37,2.05,6.33<br>7.04,7.74,7.87,10.54 |
| $HexNAc_8Hex_8Fuc_1NeuAc_1$ | 0.22,1.53,6.23,6.78,6.95<br>7.88,8.69,10.46 |
| $HexNAc_6Hex_5Fuc_3NeuAc_1$ | 0.36,1.33,2.06,2.78 |
| $HexNAc_3Hex_4Fuc_2$ | 0.0,0.28,0.77,1.31,2.31 |

*Continued on next page*

Table S1 – *Continued from previous page*

| Glycan Annotated | GUI |
|---|---|
| | 3.54,3.83,4.06,4.36,4.5 |
| | 4.84,5.08,5.73,6.16,6.66 |
| | 7.16,7.82,8.92,9.21,9.7 |
| **$HexNAc_5Hex_5Fuc_1$** | 0.2,1.29,2.81,5.54,5.94 |
| | 6.11,6.32,0.17,0.76,1.21 |
| | 2.06,5.46,5.94,6.32 |
| $HexNAc_6Hex_6Fuc_3NeuAc_3$ | 1.94,4.35,6.42,6.96,7.52 |
| | 8.61 |
| $HexNAc_7Hex_7Fuc_1$ | 0.63,1.69,2.4,2.92,5.31 |
| | 5.83,6.29,6.66,7.04,7.22 |
| | 7.39 |
| $HexNAc_9Hex_8NeuAc_1$ | 1.08,4.18,5.34,9.03 |
| $HexNAc_5Hex_5Fuc_3NeuAc_1$ | 0.38,1.78,3.92,5.65,5.87 |
| | 6.18,6.6,7.03,7.6,5.7 |
| | 6.54,7.02 |
| $HexNAc_5Hex_5Fuc_3$ | 0.53,0.89,1.53,1.78,2.28 |
| | 5.78,6.45,7.0,7.28,3.75 |
| | 5.28,6.38,6.9 |
| $HexNAc_6Hex_4$ | 0.28,1.04,5.6,0.44,1.25 |
| | 2.67,4.45,5.18,5.53,6.59 |
| **$HexNAc_4Hex_4Fuc_1NeuAc_1$** | 0.17,0.38,1.04,1.49,1.82 |
| | 2.12,2.51,5.6,5.91,6.78 |
| | 0.0,0.67,1.74,4.5,5.4 |
| | 5.64 |
| $HexNAc_9Hex_{10}Fuc_4NeuAc_1$ | 6.69 |
| $HexNAc_{10}Hex_{10}Fuc_2$ | 8.42 |
| $HexNAc_9Hex_{10}Fuc_1NeuAc_2$ | 4.18 |
| $HexNAc_9Hex_8Fuc_4NeuAc_1$ | 10.58 |
| $HexNAc_9Hex_{10}Fuc_2NeuAc_2$ | 10.63 |
| $HexNAc_{10}Hex_8Fuc_5NeuAc_1$ | 10.58 |
| $HexNAc_9Hex_{10}Fuc_3NeuAc_1$ | 10.5 |
| $HexNAc_8Hex_9Fuc_2NeuAc_1$ | 6.54 |
| $HexNAc_9Hex_{10}Fuc_1NeuAc_1$ | 8.96,10.38 |
| $HexNAc_{10}Hex_8Fuc_4NeuAc_1$ | 10.54 |
| $HexNAc_9Hex_9Fuc_2NeuAc_1$ | 9.92 |
| $HexNAc_8Hex_9Fuc_2NeuAc_2$ | 9.02,9.74 |
| $HexNAc_9Hex_9Fuc_4$ | 7.6 |
| $HexNAc_{10}Hex_9Fuc_2NeuAc_1$ | 7.44 |
